# Systematic literature review and meta-analysis of US-approved LAMA/LABA therapies versus tiotropium in moderate-to-severe COPD

**DOI:** 10.1038/s41533-018-0099-1

**Published:** 2018-08-27

**Authors:** MeiLan K. Han, Riju Ray, Jason Foo, Chaienna Morel, Beth Hahn

**Affiliations:** 1 University of Michigan, Ann Arbor, MI USA; 2 GSK Research, Triangle Park, NC USA; 3 Mapi Group, Houten, The Netherlands

**Keywords:** Chronic obstructive pulmonary disease, Outcomes research

## Abstract

Dual bronchodilator maintenance therapy may benefit patients with moderate-to-severe chronic obstructive pulmonary disease (COPD) versus long-acting muscarinic antagonist (LAMA) monotherapy. The efficacy and safety of US-approved LAMA/long-acting beta-agonist (LABA) combinations versus tiotropium (TIO), a LAMA, were assessed. This systematic review and meta-analysis (GSK: 206938), conducted in MEDLINE, MEDLINE In-process, and EMBASE following Preferred Reporting Items for Systematic reviews and Meta-Analyses guidelines, identified randomized clinical trials (>8 weeks) in moderate-to-severe COPD (per Global Initiative for Chronic Obstructive Lung Disease guidelines), receiving LAMA/LABA or TIO. Endpoints: difference in change from baseline in lung function (forced expiratory volume in 1 s [FEV_1_]; trough, peak, area under the curve 0–3 h post-dose (AUC_0–3_), St George’s Respiratory Questionnaire (SGRQ) responder rate (≥4-unit improvement), SGRQ total score, and rescue medication use at 12 and 24 weeks. Safety was also assessed. From 5683 citations, the meta-analysis included eight clinical trials. LAMA/LABA significantly improved FEV_1_ trough (Week 12: 63.0 mL, 95% confidence intervals [CI]: 39.2, 86.8; Week 24: 66.1 mL, 95% CI: 40.0, 92.3), peak (Week 12: 91.5 mL, 95% CI: 70.5, 112.4; Week 24: 92.4 mL, 95% CI: 72.9, 111.9), AUC_0–3_ (Week 12: 126.8 mL, 95% CI: 108.1, 145.4), SGRQ responder rate at Week 12 (risk ratio: 1.19; 95% CI: 1.09, 1.28), mean SGRQ total score (Week 12: −1.87, 95% CI: −2.72, −1.02; Week 24: −1.05, 95% CI: −2.02, −0.09), and rescue medication use (Week 24: −0.47 puffs/day, 95% CI: −0.64, −0.30) versus TIO (all *p* ≤ 0.03). The SGRQ responder rate at 24 weeks and adverse events were not significantly different between treatments. US-approved LAMA/LABA therapies improved lung function, SGR,Q and rescue medication use versus TIO, without compromising safety.

## Chronic lung disease: Examining dual therapy efficacy

Dual maintenance therapies combining two types of long-acting bronchodilator appear to be effective and safe for treating moderate-to-severe chronic obstructive pulmonary disease (COPD). Given that patients with COPD often have poor quality of life and, in the US, incur substantial healthcare costs, it is vital to provide optimal symptom management to improve lung function and limit exacerbations over time. Beth Hahn at GSK, North Carolina, and co-workers carried out a literature review and meta-analysis of 8 clinical trials, which indicates that combined long-acting muscarinic antagonist (LAMA)/long-acting beta-agonist (LABA) treatments—currently prescribed in the US—are an effective and safe way of tackling COPD symptoms. The team compared patients using LAMA/LABA for 12 weeks with those on single LAMA (tiotropium) treatment and found LAMA/LABA significantly improved lung function and reduced exacerbation risk.

## Introduction

Chronic obstructive pulmonary disease (COPD) is associated with chronic morbidity and mortality^1^ and accounted for 39.1 deaths per 100,000 people in the USA in 2014, according to the Centers for Disease Control and Prevention (https://www.cdc.gov/copd/data.html, accessed Aug 2017). As patients with COPD often experience a poor quality of life (QoL)^2^ and incur substantial healthcare costs^3^, determining the optimum maintenance therapy to improve their lung function, reduce the number and severity of exacerbations, improve QoL and reduce the overall costs is of primary importance.^4^ Maintenance therapy that includes a long-acting muscarinic antagonist (LAMA)/long-acting beta-agonist (LABA) combination has been recommended by Global Initiative for Chronic Obstructive Lung Disease (GOLD) 2017 as an appropriate starting treatment regimen for patients with COPD who are symptomatic and are at risk for exacerbations.^1^

The GOLD guidance has led to the increasing use of fixed-dose dual bronchodilators (LAMA/LABA) in the USA.^5^ Several network meta-analyses have demonstrated increased efficacy in terms of lung function for individual LAMA/LABA combination therapies compared with LAMA alone, with no significant difference in the number of adverse events (AEs),^5–7^ but these prior reports did not focus on the efficacy and safety of doses and formulations approved for use in the USA. Following on from these studies, the goal of this systematic review and meta-analysis is to assess the relative efficacy, as measured by lung function, heath-related QoL and rescue medication use, and the relative safety of LAMA/LABA at US-approved dosages as a class compared with TIO, as the leading LAMA monotherapy, in patients with moderate-to-severe COPD.

## Results

### Systematic literature review

**Fig. 1:**
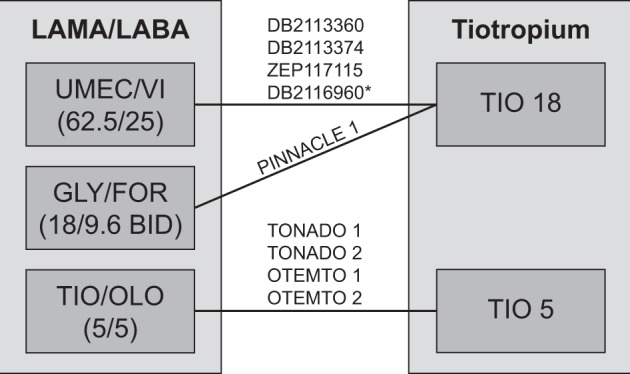
Global network of trials. *Based on the results of the feasibility assessment, the DB2116960 trial was excluded from the meta-analysis due to differences in trial design and patient characteristics. *BID* twice daily, *FOR* formoterol, *GLY* glycopyrrolate, *LABA* long-acting beta agonist, *LAMA* long-acting muscarinic antagonist, *OLO* olodaterol, *TIO* tiotropium, *UMEC* umeclidinium, *VI* vilanterol

Of the 5683 citations identified, 15 publications reporting on seven clinical trials were identified for data extraction (Figure S1). Two additional trials (DB2116960 and PINNACLE 1), not identified in the original search as they were published after the search date, were also included. The search of the clinical trial registries identified nine trials for inclusion. Of these nine trials, eight were matched to trials identified in the literature search and one was an ongoing trial (DB204990; NCT02799784). All trials included in this meta-analysis compared TIO 5 or 18 µg with LAMA/LABA; there were no trials identified that compared GLY/IND 15.6/27.5 µg BID with TIO (Fig. 1). Inhaled corticosteroids (ICS) therapy was allowed in all trials, with ICS use at baseline ranging from 33.7% (PINNACLE 1) to 54.4% (ZEP117115) of patients.

### Critical appraisal and feasibility assessment

Overall, the risk-of-bias assessment demonstrated that most trials included a low risk of bias as they used randomization generation, allocation concealment, and blinding for both trial participants and caregivers (Table 1). However, PINNACLE 1, which included an open-label TIO arm, was assigned a high risk of bias due to inadequate blinding of participants, personnel, and outcome assessors. In addition, the blinding of the outcome assessors and handling of missing data were not described in half of the trials.

**Table 1:** Level of bias of included studies

	Random sequence generation	Allocation concealment	Blinding of participants and personnel	Blinding of outcome assessors	Incomplete outcome data	Selective reporting	Other bias
DB2113360	Low	Low	Low	Low	Low	Low	Low
DB2113374	Low	Low	Low	Low	Low	Low	Low
ZEP117115	Low	Low	Low	Low	Low	Low	Low
DB2116960	Low	Low	Low	Low	Low	Low	Low
PINNACLE 1	Low	Low	High	High	Unclear	Low	Low
TONADO 1	Low	Low	Low	Unclear	Unclear	Low	Unclear
TONADO 2	Low	Low	Low	Unclear	Unclear	Low	Unclear
OTEMTO 1	Unclear	Unclear	Low	Unclear	Unclear	Low	Low
OTEMTO 2	Unclear	Unclear	Low	Unclear	Unclear	Low	Low

Following a feasibility assessment, one trial (DB2116960) was excluded as an outlier based on differences in trial design and patient characteristics (milder COPD population not receiving ICS therapy; Table 2 and supplementary materials [Table S3]). Therefore, eight trials were included in the analysis. The excluded trial was included in a scenario analysis which demonstrated results consistent with the base case (data not shown).

**Table 2:** Study design and patient characteristics identified from the systematic literature review (arms of interest only)

Study	Treatments (ITT)	Trial duration (weeks)	Male (%)	Age, y (SD)	Current smoker (%)	Severe or very severe^a^ (%)	ICS use (%)	Pack years (SD)	Mean FEV1^b^ (L)	FEV_1_ % predicted (SD)
DB2113360^12^	UMEC/VI; 62.5/25 μg; OD (*n* = 212)	24	69.8	63 (8.7)	46.2	50.5	43.9	44.8 (27.7)	1.31 (0.487)	48 (12.9)
	TIO; 18 μg; OD (*n* = 208)		67.3	62.6 (9.4)	47.6	52.9	44.7	41.9 (24.4)	1.30 (0.502)	47.8 (13.4)
DB2113374^12^	UMEC/VI; 62.5/25 μg; OD (*n* = 217)	24	64.5	65.0 (8.6)	42.4	50.7	47.5	47.8 (26.1)	1.17 (0.466)	47.7 (13.5)
	TIO; 18 μg; OD (*n* = 215)		71.2	65.2 (8.3)	47.4	51.6	53.5	54.0 (31.6)	1.18 (0.429)	47.4 (13.1)
ZEP117115^13^	UMEC/VI; 62.5/25 μg; OD (*n* = 454)	24	68.3	61.9 (8.4)	59.5	59.3	54.4	44.1 (24.4)	1.26 (0.460)	46.2 (13.0)
	TIO; 18 μg; OD (*n* = 451)		67.2	62.7 (8.5)	53.7	57.9	52.5	44.4 (25.0)	1.26 (0.477)	46.5 (12.8)
DB2116960^19^	UMEC/VI; 62.5/25 μg; OD (*n* = 247)	12	66.0	64.5 (8.7)	52.2	11.3	NR	38.6 (20.5)	NR	59.8 (5.5)
	TIO; 18 μg; OD (*n* = 247)		64.8	64.3 (8.7)	47.8	13.8	NR	40.4 (20.2)	NR	59.4 (5.3)
PINNACLE 1^20^	GLY/FOR; 18/9 μg; BID (*n* = 526)	24	55.1	62.6 (8.4)	53.4	46.0	33.7	50.9 (26.8)	NR	51.4 (13.6)
	TIO; 18 μg; OD (*n* = 451)		59.6	63.0 (8.6)	52.8	47.2	36.4	53.0 (27.5)	NR	51.4 (13.8)
TONADO 1^8^	TIO/OLO; 5/5 μg; OD (*n* = 522)	52	73.6	64.8 (8.2)	36.2	50.6	51.7	NR	1.17 (0.47)	49.5 (15.2)
	TIO; 5 μg; OD (*n* = 527)		72.7	64.2 (8.5)	35.7	50.1	45.0	NR	1.20 (0.50)	49.7 (15.3)
TONADO 2^8^	TIO/OLO; 5/5 μg; OD (*n* = 507)	52	68.8	62.7 (8.4)	41.6	51.8	46.5	NR	1.19 (0.51)	49.1 (15.4)
	TIO; 5 μg; OD (*n* = 506)		73.5	63.5 (8.7)	36.0	49.6	45.3	NR	1.20 (0.51)	49.7 (16.1)
OTEMTO 1^21^	TIO/OLO; 5/5 μg; OD (*n* = 203)	12	56.2	64.7 (8.9)	54.7	36.0	41.9	NR	1.32 (0.491)	54.9 (12.0)
	TIO; 5 μg; OD (*n* = 203)		61.1	64.9 (8.2)	48.3	37.0	37.9	NR	1.31 (0.458)	54.7 (12.8)
OTEMTO 2^21^	TIO/OLO; 5/5 μg; OD (*n* = 202)	12	65.8	65.2 (8.5)	45.5	38.1	35.6	NR	1.36 (0.467)	54.8 (12.8)
	TIO; 5 μg; OD (*n* = 203)		64.0	64.7 (8.4)	44.8	32.5	35.0	NR	1.40 (0.511)	55.9 (12.2)

*BID* twice daily, *FEV*_*1*_ forced expiratory volume in 1 s, *FOR* formoterol, *GLY* glycopyrrolate, *ICS* inhaled corticosteroids, *ITT* intent-to treat population, *NR* not reported, *OD* once daily, *OLO* olodaterol, *SD* standard deviation, *TIO* tiotropium, *UMEC* umeclidinium

^a^ Calculated severe and very severe patients ^b^Pre-bronchodilator

### Meta-analysis: efficacy and safety

FEV_1_ trough at 12 and 24 weeks were the most heterogeneous endpoints (*I* ^2^: 71–74%, *p* < 0.01) and were analyzed using a random effects meta-analysis model. The remaining endpoints were analyzed using a fixed effect meta-analysis model (Table S4).

### FEV_1_ trough

**Fig. 2:**
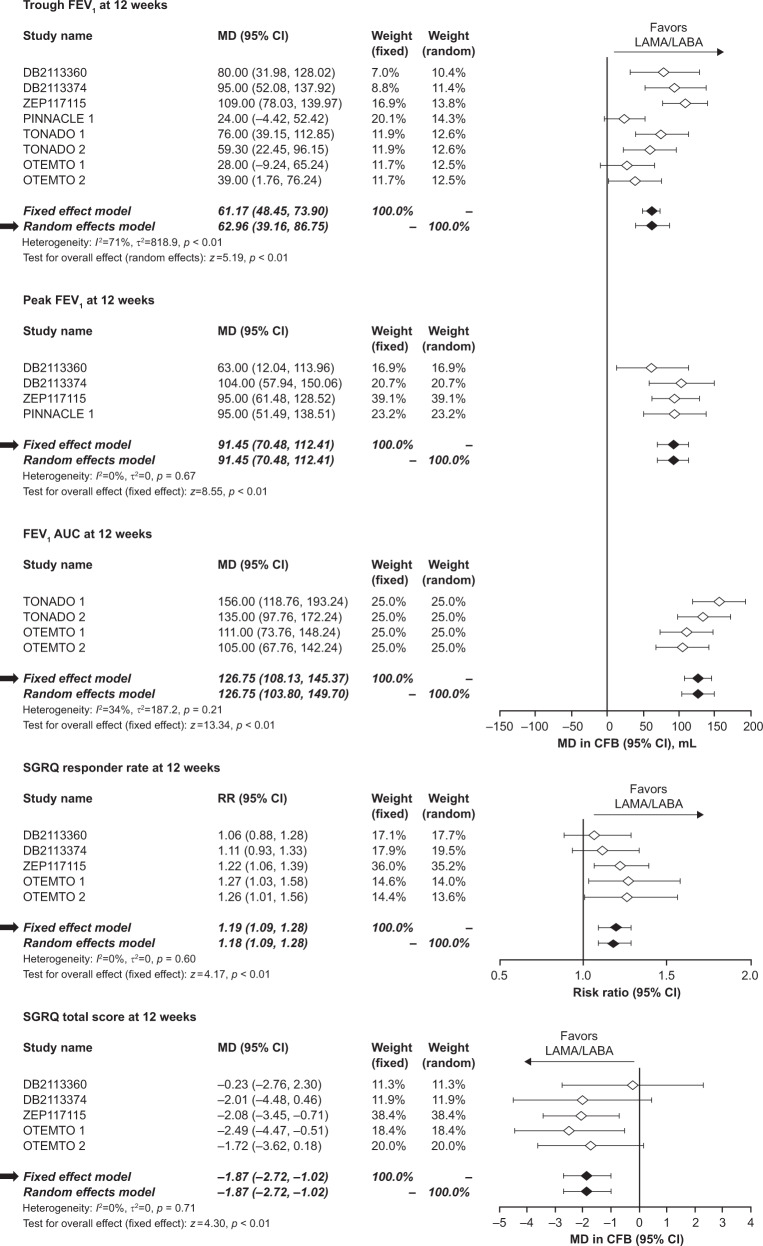
Forest plots of LAMA/LABA versus TIO at 12 weeks. *AUC* area under curve, *CFB* change from baseline, *CI* confidence interval, *FEV*_*1*_ forced expiratory volume in 1 s, *I* ^2^ proportion of variability across trials due to heterogeneity, *LABA* long-acting beta agonist, *LAMA* long-acting muscarinic antagonist, *MD* mean difference, *RR* risk ratio, *SGRQ* St Georges Respiratory Questionnaire, *τ* ^2^ between trial variance in random effects meta-analysis, *TIO* tiotropium

**Fig. 3:**
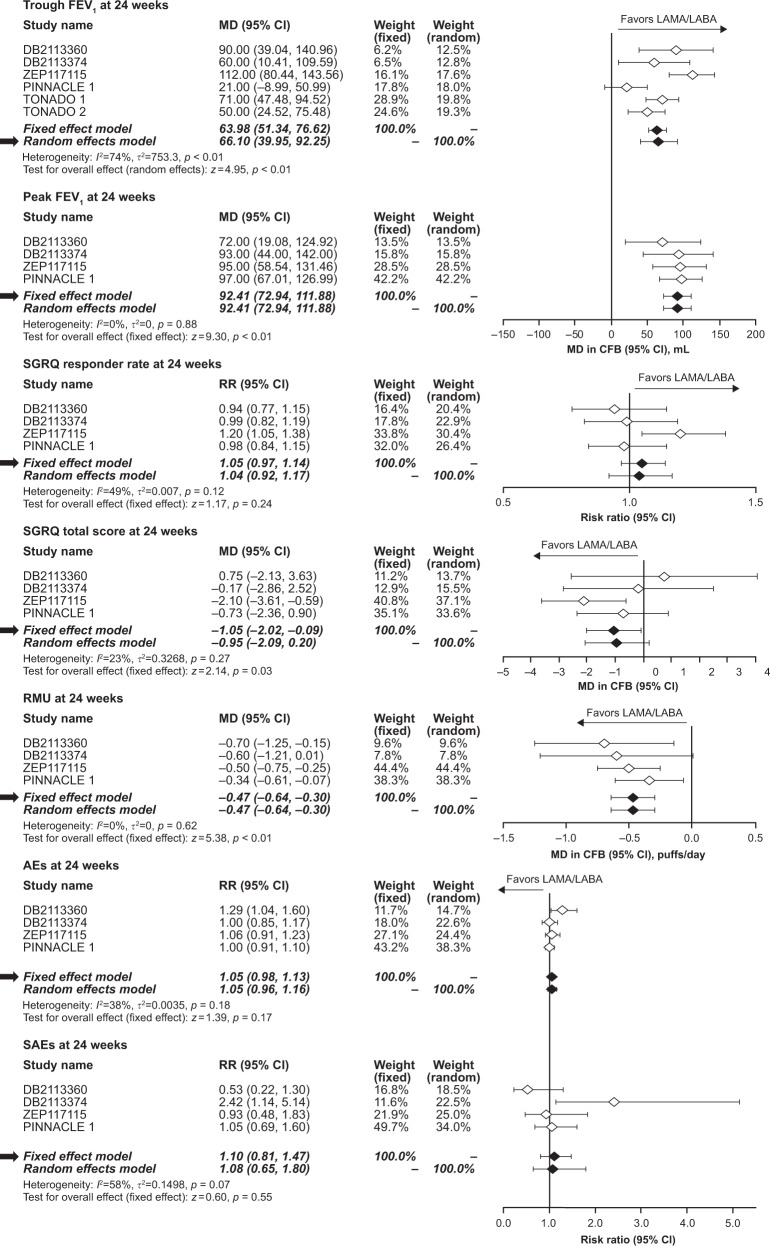
Forest plots of LAMA/LABA versus TIO at 24 weeks. *AE* adverse event, *CFB* change from baseline, *CI* confidence interval, *FEV*_*1*_ forced expiratory volume in 1 s, *I* ^*2*^ proportion of variability across trials due to heterogeneity, *LABA* long-acting beta agonist, *LAMA* long-acting muscarinic antagonist, *MD* mean difference, *RMU* rescue medication use, *RR* risk ratio, *SAE* serious adverse event, *SGRQ* St Georges Respiratory Questionnaire, *τ* ^*2*^ between study variance in random effects meta-analysis, *TIO* tiotropium

At 12 weeks, the meta-analysis demonstrated that LAMA/LABA treatment significantly improved FEV_1_ trough by 63.0 mL (95% confidence intervals [CI]: 39.2, 86.8) compared with TIO (*p* < 0.01). At 24 weeks, the improvement remained significant (66.1 mL; 95% CI: 40.0, 92.3; *p* < 0.01) (Table 3, Figs. 2 and  3).

**Table 3:** Overview of all meta-analysis outcomes

	**Outcome**	**LAMA/LABA versus TIO results (95% CI)** ^†^
	**12 weeks**	
	FEV_1_ trough; ΔCFB (mL)	62.96 (39.16; 86.75)**
	FEV_1_ peak; ΔCFB (mL)	91.45 (70.48; 112.41)**
	FEV_1_ AUC; ΔCFB (mL)	126.75 (108.13; 145.37)**
	SGRQ responder rate; risk ratio	1.19 (1.09; 1.28)**
	SGRQ total score; ΔCFB	−1.87 (−2.72; −1.02)**
	RMU; ΔCFB (puffs/day)	N/A
	AEs; risk ratio	N/A
	SAEs; risk ratio	N/A
	**24 weeks**	
	FEV_1_ trough; ΔCFB (mL)	66.10 (39.95; 92.25)**
	FEV_1_ peak; ΔCFB (mL)	92.41 (72.94; 111.88)**
	FEV_1_ AUC; ΔCFB (mL)	N/A
	SGRQ responder rate; risk ratio	1.05 (0.97; 1.14)
	SGRQ total score; ΔCFB	−1.05 (−2.02; −0.09)*
	RMU; ΔCFB (puffs/day)	−0.47 (−0.64; −0.30)**
	AEs; risk ratio	1.05 (0.98; 1.13)
	Exploratory analyses	1.03 (0.99; 1.07)^‡^
	SAEs; risk ratio	1.10 (0.81; 1.47)
	Exploratory analyses	1.02 (0.87; 1.20)^‡^

*AE* adverse event, *AUC* area under curve, *ΔCFB* difference in change from baseline; *CI* confidence interval, *FEV*_*1*_ forced expiratory volume in 1 s, *LABA* long-acting beta agonist, *LAMA* long-acting muscarinic antagonist, *N/A* not applicable, *RMU* rescue medication use, *SAE* serious adverse event, *SGRQ* St Georges Respiratory Questionnaire, *TIO* tiotropium

* *p* < 0.05 **p < 0.01 ^†^Results are shown for either the fixed effect model (*p-*value for heterogeneity test is ≥0.05) or the random effects model (*p-*value for heterogeneity test is <0.05) ^‡^Exploratory analysis including TONADO 1 and TONADO 2 at 52 weeks

### FEV_1_ peak

Compared with TIO, the meta-analysis demonstrated that LAMA/LABA treatment was associated with significant improvements in FEV_1_ peak at both 12 and 24 weeks, with a pooled effect size of 91.5 mL (95% CI: 70.5, 112.4) and 92.4 mL (95% CI: 72.9, 111.9), respectively (*p* < 0.01; Table 3, Figs. 2 and  3).

### FEV_1_ AUC_0–3_

For FEV_1_ AUC_0–3_, the meta-analysis demonstrated that dual bronchodilation with LAMA/LABA provided a significant improvement of 126.8 mL (95% CI: 108.1, 145.4) at 12 weeks versus TIO (*p* < 0.01; Table 3, Fig. 2). No meta-analysis was conducted for FEV_1_ AUC at Week 24 as data were only reported in TONADO 1 and 2. However, published pooled results showed a significant improvement of 110 mL for LAMA/LABA versus TIO.^8^

### SGRQ responder rate

At 12 weeks, LAMA/LABA demonstrated a 19% greater SGRQ responder rate than TIO (risk ratio [RR]: 1.19, 95% CI: 1.09, 1.28; *p* < 0.01). The SGRQ responder rates at 12 weeks ranged from 51.8% to 58.7% for LAMA/LABA combinations and from 41.1% to 53.2% for TIO across all trials (Table S5). At 24 weeks, LAMA/LABA was associated with a 5% greater responder rate than TIO (RR: 1.05, 95% CI: 0.97, 1.14); however, the effect was not significant (*p* = 0.24; Table 3, Figs. 2 and 3). The SGRQ responder rates at 24 weeks ranged from 37.6% to 54.2% for LAMA/LABA combinations and from 38.4% to 54.7% for TIO across all trials (Table S5).

### SGRQ total score

The meta-analysis demonstrated that LAMA/LABA significantly improved SGRQ total score at 12 weeks compared with TIO (improvement of 1.87 points, 95% CI: −2.72, −1.02; *p* < 0.01). Similar results were observed at 24 weeks (improvement of 1.05 points, 95% CI: −2.02, −0.09; *p* = 0.03; Table 3, Figs. 2 and 3).

### Rescue medication use

There were no trials that reported rescue medication use at 12 weeks, so no meta-analysis was performed for this time point. At 24 weeks, rescue medication use was significantly reduced by 0.47 puffs/day (95% CI: −0.64, −0.30; *p* < 0.01) in patients receiving LAMA/LABA compared with those receiving TIO (Table 3, Fig. 3).

### Safety

At 24 weeks, treatment with LAMA/LABAs did not significantly affect the proportion of patients experiencing AEs (RR: 1.05, 95% CI: 0.98, 1.13, *p* = 0.17) or SAEs (RR: 1.10, 95% CI: 0.81, 1.47, *p* = 0.55) compared with TIO (Table 3, Fig. 3). In addition, an exploratory analysis, which also included 52-week data from the TONADO 1 and 2 trials with the 24-week data, also demonstrated that there was no significant difference between the two treatments in the proportion of patients experiencing AEs (RR: 1.03, 95% CI: 0.99, 1.07, *p* = 0.19) or SAEs (RR: 1.02, 95% CI: 0.87, 1.20, *p* = 0.81) (Table 3, Figure S2).

The results of the meta-analyses for each endpoint were broadly consistent with the individual trial results (Table S5).

## Discussion

The results presented here demonstrate that 12 weeks of treatment with LAMA/LABA significantly improves lung function (FEV_1_ trough, FEV_1_ peak) and QoL (SGRQ responder rate and total score) compared with TIO. Data from studies examining 24 weeks of treatment demonstrated similar improvements in FEV_1_ trough, FEV_1_ peak, and SGRQ total score in addition to improvements in rescue medication use, although significant improvements in SGRQ responder rates with LAMA/LABA versus TIO were not maintained at 24 weeks. The present analysis also demonstrates that dual bronchodilation with LAMA/LABA therapy was not associated with any additional safety concerns compared with LAMA treatment alone, as there were similar proportions of patients with AEs who were receiving LAMA/LABA or TIO.

GOLD 2017 currently recommends that symptomatic patients with COPD can be initially treated with a LAMA, and then stepped up to receive a LAMA/LABA combination if they remain symptomatic.^1^ In addition, patients with COPD experiencing severe breathlessness may also be recommended a LAMA/LABA combination as the first line of treatment.^1^ Our meta-analysis demonstrated improvements in lung function, reduced rescue medication use, and a similar safety profile with dual bronchodilator therapy as compared with LAMA monotherapy with TIO. Our results are consistent with two previous meta-analyses showing that LAMA/LABA treatment significantly improved lung function and QoL without significantly affecting the safety profile versus LAMA in patients with stable or moderate-to-severe COPD.^5,7^ These studies included similar LAMA/LABA combinations to this meta-analysis and included 3–4 of the same studies, which may explain some of the consistency between the findings. One of these previous meta-analyses showed that LAMA/LABA combinations (UMEC/VI 62.5/25 and 125/25 μg, IND/GLY 110/50 μg, TIO/IND 18/150 μg, and TIO/OLO 5/5, 18/5, and 2.5/5 μg) improved lung function and SGRQ scores from 3 to 12 months versus LAMA.^7^ The second meta-analysis demonstrated that LAMA/LABA combinations of UMEC/VI 62.5/25 μg, IND/GLY 27.5/15.6 and 110/50 μg, and TIO/OLO 5/5 μg also improved FEV_1_ trough (12, 24–26, and 52 weeks), FEV_1_ peak (12 and 24–26 weeks), SGRQ total scores (12 and 24 but not 52 weeks), and rescue medication use (12–64 weeks) versus LAMA in patients with moderate-to very severe COPD.^5^ In the present study, the reason why the significant improvements in SGRQ responder rates observed at 12 weeks were not maintained at 24 weeks is unclear. Likewise, our findings are in-keeping with a 2015 Cochrane review which addressed the relative effects on markers of QoL, lung function, symptoms, and SAEs in patients with moderate-to severe COPD randomized to LABA + TIO versus either TIO or LABA alone.^9^ Like our study, this review demonstrated that compared with TIO alone, LABA + TIO resulted in improvements in FEV_1_ as well as in SGRQ score, although between-treatment differences did not always reach the MCID (100 mL for FEV_1_ and 4 units for SGRQ). However, similar to the present study, a SGRQ responder analysis indicated that 7% more participants receiving LABA + TIO had a clinically meaningful benefit compared with TIO therapy alone.

The main strength of this study is that it provides important information about the use of LAMA/LABA combination therapies at US-approved doses, a topic which to date has had limited attention. However, our study is not without limitations. One of the limitations is that TIO was the only LAMA monotherapy considered as a comparator to the dual bronchodilators, of which only one contains TIO as a component. TIO was selected as it was the most commonly used LAMA monotherapy in the USA, representing over 70% of the entire bronchodilator market at the time the literature search was conducted,^10^ and was therefore considered to be the most appropriate choice of comparator in this study. An additional limitation of this study was that no trials were identified that compared GLY/IND 15.6/27.5 µg BID, one of the four US-approved LAMA/LABA therapies, with TIO, which may limit the generalizability of the conclusions to all LAMA/LABA therapies approved for COPD in the US. However, as previous studies have demonstrated that GLY/IND 15.6/27.5 µg BID may provide significant improvements in lung function versus GLY or IND,^11^ it is likely that that GLY/IND 15.6/27.5 µg BID would perform better than TIO at improving lung function. Finally, additional patient-important outcomes such as exacerbation events and hospitalizations were not evaluated in this meta-analysis. These may have been interesting outcomes to highlight the potential clinical implications of treatment with LAMA/LABA therapy compared with TIO and would benefit from further study.

As the majority of published studies, including the studies mentioned above, use non-US-approved doses of LAMA/LABA (IND/GLY 110/50 μg^7^ and TIO/IND 18/150 μg^7^), it is difficult to assess the efficacy of LAMA/LABA combinations versus LAMA in patients with COPD in the USA. Based on the limited studies conducted using LAMA/LABA at US-approved doses, future treatment strategies in COPD would benefit from further studies comparing the number of exacerbations between US-approved LAMA/LABA therapies and LAMA monotherapy in patients with moderate-to very severe COPD. Previous studies conducted using US-approved doses of UMEC/VI 62.5/25 μg have demonstrated that the time to first exacerbation was similar compared with LAMA or TIO in patients with moderate-to-severe COPD.^12^ However, the percentage of exacerbations were lower with UMEC/VI 62.5/25 μg treatment versus TIO.^13^

Although our study has demonstrated the benefits of LAMA/LABA dual bronchodilation versus TIO, another meta-analysis has indicated that LAMA/LABA treatment may also provide improvements in lung function and rescue medication use but not SGRQ score over LABA/ICS.^5^ Further research is needed to examine why the improvements in lung function did not translate into sustained improvements in SGRQ response.

Overall, this meta-analysis demonstrates that treatment with LAMA/LABA improves lung function, reduces symptoms, and improves QoL compared with TIO, without significantly increasing the risk of AEs in patients with moderate-to-severe COPD. These results support the current objectives of GOLD 2017 and COPD foundation 2017 guidelines, which are to reduce the impact of symptoms^1,14^ and reduce the risk of AEs in patients with COPD.^1^

## Materials and methods

### Systematic literature review

This was a systematic literature review (GSK protocol: 206938) following Preferred Reporting Items for Systematic reviews and Meta-Analyses (PRISMA) guidelines, to identify randomized clinical trials of >8 weeks duration in patients with moderate-to-severe COPD. The primary search was conducted in MEDLINE, MEDLINE In-process, and EMBASE, with additional searches of the Cochrane Central Register of Controlled Trials (Cochrane CENTRAL), Cochrane Database of Systematic Reviews (Cochrane CDSR), and Database of Abstracts of Reviews of Effects (DARE). The search strategies combined ‘disease terms’ with ‘trial design terms’ and were restricted to the English language with no time restrictions up until the search date (November 29, 2016). The search strategies used for the systematic literature review are included in Table S1. Each trial was screened for relevance by a researcher and confirmed by another, based on the eligibility criteria described in Table S2.

Additionally, two clinical trial registries were searched to identify planned or ongoing studies: clinicaltrials.gov (National Institute of Health) from the USA and the Clinical Trials Registry Platform Search Portal (ICTRP) from the World Health Organization (WHO). No time restrictions were used for the searches. Studies identified through the search of clinical trial registries were cross-checked against those identified from the systematic literature review.

After trials had been identified, the key trial design details, patient characteristics, and outcomes were extracted from tables, text, and figures. DigitizeIt software was used to extract data from figures. Data extraction was performed by one researcher and checked by another. Inconsistencies were discussed between the researchers or with a third, independent researcher.

### Endpoints

Endpoints included difference in change from baseline (ΔCFB) in forced expiratory volume in 1 s (FEV_1_) trough, peak, and area under the curve at 0–3 h post-dose (AUC_0–3_) at 12 and 24 weeks. FEV_1_ was chosen as an endpoint as it assesses lung function by measuring the volume of air, usually in mL or L, expelled by the lungs within 1 s after the lungs have been filled by taking a deep breath. Lower FEV_1_ values are associated with more airway obstruction or bronchoconstriction. FEV_1_ trough refers to the FEV_1_ calculated at the end of a 24-h dosing interval, whereas peak FEV_1_ is the maximum value observed after drug administration. The AUC is calculated from the FEV_1_–time curve, which is plotted using measurements taken pre- and post-dose.

St George’s Respiratory Questionnaire (SGRQ) responder rate and the ΔCFB in SGRQ total score was also evaluated at 12 and 24 weeks. The SGRQ is a respiratory-specific QoL patient-reported outcome (PRO) tool that assesses the domains of symptoms, activity, and impact on health status in COPD, with lower total scores indicating improved health status.^15^ The SGRQ responder rate describes the percentage of patients with at least a minimal clinically important difference (MCID) of a 4-unit reduction from baseline in SGRQ total score.^16^

Additional endpoints included ΔCFB in rescue medication use, as measured by the number of puffs per day at 12 and 24 weeks, the proportion of patients reporting AEs and serious AEs (SAEs) at 24 weeks. No meta-analysis was conducted for AEs or SAEs at 12 weeks, as we considered this too short a time point to provide the most clinically meaningful results for safety. For each time point, trials were included if they reported these endpoints within a margin of 4 weeks (8–16 and 20–28 weeks, respectively).

### Treatments

Trials that were identified were used to compare TIO 5 µg once daily (OD) (Respimat) or TIO 18 µg OD (Handihaler) with umeclidinium (UMEC)/vilanterol (VI) 62.5/25 µg OD (Anoro), olodaterol (OLO)/TIO 5/5 µg OD (Stiolto), glycopyrrolate (GLY)/formoterol (FOR) 18/9.6 µg twice daily (BID) (Bevespi), and GLY/indacaterol (IND) 15.6/27.5 µg BID (Utibron).

### Critical appraisal and feasibility assessment

The trial design and methodology of all identified trials were critically appraised using the Cochrane risk-of-bias assessment questionnaire.^17^ This questionnaire assessed whether trials were at risk of bias based on the level of blinding, allocation concealment, randomization methods, selective reporting, and completeness of outcome data.

A feasibility analysis was performed to evaluate the possibility of performing a meta-analysis of LAMA/LABA versus TIO in patients with moderate-to-severe COPD. Across the trials, the analysis assessed differences in trial design and patient characteristics which would affect the comparative treatment effects, the availability and comparability of data reported, and the specificities of the evidence base identified. The assessment allowed for the selection of trials with similar treatment duration and dosages for analysis.

### Statistical analysis

Risk ratios of LAMA/LABA over TIO were calculated for SGRQ responder rates, AEs, and SAEs. Values of >1 (SGRQ responder rate) or <1 (AEs and SAEs) indicated more favorable results for LAMA/LABA treatment versus TIO.

The statistical approach was prospectively planned and both fixed and random effects models were employed for the meta-analysis; model selection was data-driven. The fixed effect model assumed that each trial measured the same parameter with no variation in the source population, whereas the random effects model allowed trial outcomes to vary in a normal distribution between trials. Clinical and methodological heterogeneity was determined using *I* ^2^, which is a statistic that expresses the inconsistency of results between studies. Depending on the outcome type, different statistical methods were used to analyze the meta-analysis results. These included the inverse variance method (T^2^) for continuous outcomes and the Mantel–Haenszel methods for dichotomous outcomes. The Mantel–Haenszel method is generally preferable to the inverse variance for dichotomous outcomes; however, it often results in similar estimates to the inverse variance method.^17^ Further analysis details are included in the supplementary materials. Analyses were conducted using R software^©^ version 1.0.44 (RStudio, Inc., Boston, MA, USA) and meta and metafor meta-analysis packages.^18^

## Electronic supplementary material

Supplementary Material

## Data Availability

All data generated or analyzed during this study are included in this published article (and its supplementary information files). Information on GSK’s data sharing commitments and requesting access can be found at www.clinicalstudydatarequest.com. GSK (Study number: 206938)

## Electronic supplementary material

**Supplementary information** accompanies the paper on the *npj Primary Care Respiratory Medicine* website (10.1038/s41533-018-0099-1).
