# Author Correction: Systematic literature review and meta-analysis of US-approved LAMA/LABA therapies versus tiotropium in moderate-to-severe COPD

**DOI:** 10.1038/s41533-021-00264-6

**Published:** 2021-12-15

**Authors:** MeiLan K. Han, Riju Ray, Jason Foo, Chaienna Morel, Beth Hahn

**Affiliations:** 1 University of Michigan, Ann Arbor, MI USA; 2 GSK Research, Triangle Park, NC USA; 3 Mapi Group, Houten, The Netherlands

Correction to: *npj Primary Care Respiratory Medicine* 10.1038/s41533-018-0099-1, published online 27 August 2018

In this article the ‘Competing Interests’ statement was incomplete and should have included ‘M.K.H. reports consulting fees from Boehringer Ingelheim, GSK and AstraZeneca. She also reports research support from Novartis and Sunovion. GSK also supported publication of this article.’ The original article has been corrected.

