# Supplementary material for: Systematic literature review and meta-analysis of US-approved LAMA/LABA therapies versus tiotropium in moderate-to-severe COPD
Source: NPJ Prim Care Respir Med. 2018 Aug 27;28:32. doi: 10.1038/s41533-018-0099-1 (PMC6110857; doi:10.1038/s41533-018-0099-1)
Supplement: Supplementary file 1 — Supplementary Material [file 41533_2018_99_MOESM1_ESM.docx]

# eSupplementary materials

# Methods

## Statistical analysis

### Heterogeneity

Clinical (patient characteristics) and methodological (study design) heterogeneity was determined using Cochran’s Q and I^2^. Cochran’s Q was calculated as the weighted sum of squared differences between individual study effects and the pooled effects across studies, with the weights being those used in the pooling method. I^2^ was calculated using I² = 100% x (Q-df)/Q, where df are the degrees of freedom. I^2^ assessed the percentage of variation due to heterogeneity compared with chance, with an I^2^ of 0–25% indicating a mild, acceptable heterogeneity, 25–50% indicating a moderate heterogeneity and >50% indicating high heterogeneity.

Features of the study design that were assessed included inclusion criteria (related to forced expiratory volume in 1 second [FEV_1_], FEV_1_/forced vital capacity [FVC], exacerbations, and smoking), additional COPD treatment (inhaled corticosteroids [ICS] and/or LABA), randomization, blinding, open-label arms and cross over design. For baseline patient characteristics, exacerbation history, percentage of patients per chronic obstructive pulmonary disease (COPD) severity level, mean FEV_1_ % predicted, percentage of current smokers, mean pack-years, percentage of male patients, and mean age were assessed.

Given the relatively low number of studies, meta-regression was not performed. Instead, analysis excluding evidence where clinical heterogeneity exists was conducted.

### Meta-analysis models

The fixed effect model assumes that all studies share a common true effect size. The summary statistic Y_i_, where i=1,2,…,k independent studies, is normally distributed with E(Y_i_)=θ, where θ is the common true effect size and E is the expected mean. The variance of Y_i_ was calculated using s_i_^2^=var(Y_i_), where s_i_^2^ is known.

The random effects model assumes that the studies are drawn from populations that differ from each other, potentially impacting the relative treatment effect. The summary statistic Y_i_, where i=1,2,…,k independent studies, is normally distributed with Y_i_| θ_i_, s_i_^2^ ~ Normal (θ_i_, s_i_^2^), where θ_i_ represents the unique true effect size in the i^th^ study and s_i_^2^ represents the estimated within-study variance for the i^th^ study. The model assumes that the study-specific mean θ_i_ is drawn from the following population: θ_i_|µ, τ^2^ ~ Normal (µ, τ^2^), where the parameters µ and τ^2^ represent the overall pooled true effect size and the between-study variance, respectively.

### Statistical models

Depending on the outcome type, different statistical methods were used to analyze the meta-analysis results. These included the inverse variance method (T^2^) for continuous outcomes and the Mantel-Haenszel methods for dichotomous outcomes. The Mantel-Haenszel method is generally preferable to the inverse variance for dichotomous outcomes; however, it often results in similar estimates to the inverse variance method.^1^ For studies that did not report any measure of uncertainty (standard error [SE] or standard deviation [SD]), an imputation of the SE was made using the average SD of the other studies included in the meta-analysis of the same outcome. The robustness of the meta-analysis results with imputation was assessed using the minimum and maximum SD.

### Treatments and outcomes analyzed

FEV_1_ (peak, trough, and AUC), SGRQ (change from baseline), and rescue medication were expected to generate normally distributed continuous data. SGRQ responder rate and adverse events/serious adverse events are expected to generate dichotomous data. For the individual trials, continuous data were reported as point estimates and 95% confidence intervals (CI) versus dichotomous data, which were reported as number with the event, number in the group, and risk ratio. The pooled results were also reported as point estimates and 95% CI.

# Results

## Feasibility assessment

### Trial design

The trial design and patient characteristics were similar for all trials except for DB2116960, which had different exclusion criteria compared with the other trials, and PINNACLE 1, which included an open-label treatment arm for TIO (**Table S3**). All trials included >200 patients per treatment arm and did not allow additional treatment with LABA. Most trials included patients who were >40 years old, with a diagnosis of moderate-to-severe COPD and who were also receiving ICS therapy. In the DB2116960 trial, patients were >40 years old but had been diagnosed with moderate COPD and were not receiving ICS therapy. Based on the differences in trial design and patient characteristics (milder COPD population not receiving ICS therapy), the DB2116960 trial was excluded as an outlier, but was included in a scenario analysis.

### Patient characteristics

For the eight similar trials (excluding DB2116960), patients were mostly male (55.1–73.6%) with a mean age of 61.9–65.2 years. Approximately half of patients were current smokers (35.7–59.5%) with a mean FEV_1_ trough of 1.17–1.40 L. Patients with severe and very severe COPD ranged from 32.5% to 59.3% per treatment (**Table S3**).

### Outcomes

ΔCFB in FEV_1_ trough was reported by all eight trials at 12 weeks and by all but two trials (OTEMTO 1 and 2) at 24 weeks. ΔCFB in peak FEV_1_ was reported by four trials (DB2113374, DB2113360, ZEP117115, and PINNACLE 1) at 12 and 24 weeks. FEV_1_ AUC_0–3_ was reported by four trials at 12 weeks (OTEMTO 1 and 2, TONADO 1 and 2) and only by TONADO 1 and 2 at 24 weeks. SGRQ responder rate and ΔCFB in SGRQ total score were reported by five trials (DB2113374, DB2113360, ZEP117115, OTEMTO 1 and 2) at 12 weeks and four trials at 24 weeks (DB2113374, DB2113360, ZEP117115, and PINNACLE 1). ΔCFB in rescue medication use (puffs/day) was only reported at 24 weeks by four trials (DB2113374, DB2113360, ZEP117115, and PINNACLE 1). As AEs and SAEs were reported for the overall trial duration, safety data were available at three time points: 12 (OTEMTO 1 and 2), 24 (DB2113360, DB2113374, ZEP117115, PINNACLE 1) and 52 weeks (TONADO 1 and 2).

### Feasibility assessment

From the 8 trials that were deemed to be sufficiently homogeneous in trial design and patient characteristics, it was feasible to perform a meta-analysis on the following 12 of the 16 predefined outcomes: FEV_1_ trough, FEV_1_ peak, SGRQ responder rate and SGRQ total score at 12 and 24 weeks, FEV_1_ AUC_0-3h_, rescue medication use (puffs/day), AEs, and SAEs at 24 weeks. In addition, as the TONADO 1 and 2 trials only reported safety data at 52 weeks, these data were included in an exploratory analysis with the safety data reported at 24 weeks.

# Tables and Figures

## Table S1: Search strategy for the systematic literature review in Medline and MEDLINE in-Process, EMBASE, Cochrane CENTRAL, Cochrane CDSR, and DARE

| **Database:** MEDLINE® and MEDLINE® In-Process & Other Non-Indexed Citations  **Platform:** Ovid  **Date of search:** November 29, 2016  **Time Limits:** n/a  **Filters:** Lines 6– 15 are from the search filter: Cochrane Highly Sensitive Search Strategy for  identifying randomized trials in MEDLINE: sensitivity- and precision maximizing version (2008 revision); Ovid format. Available from:  http://handbook.cochrane.org/chapter_6/box_6.4.d_cochrane_hsss_2008_sensprec_ ovid.htm (accessed on October 2, 2015) | | |
| --- | --- | --- |
| **#** | **Searches** | **Hits** |
| 1 | (formoterol or eformoterol or foradil or oxis or atimos modulite or atock or perforomist or salmeterol or serevent or tiotropium or spiriva or Ba 679 BR or indacaterol or onbrez or arcapta or NVA-237 or NVA237 or (NVA adj "237") or glycopyrronium or glycopyrrolate or seebri or enurev breezhaler or aclidinium or tudorza pressair or eklira genuair or symbicort or advair or seretide or olodaterol or striverdi or umeclidinium or GSK573719 or vilanterol or GW642444 or QVA149 or relovair or zephyr or anoro ellipta).ti,ab,nm. | 7039 |
| 2 | exp Pulmonary Disease, Chronic Obstructive/ or exp Chronic obstructive lung disease/ | 47593 |
| 3 | (COPD or chronic obstructive pulmonary disease or COAD or chronic obstructive airway disease or chronic obstructive lung disease or chronic bronchitis or emphysema).ti,ab. | 77451 |
| 4 | 2 or 3 | 88137 |
| 5 | 1 and 4 | 2374 |
| 6 | randomized controlled trial.pt. | 469809 |
| 7 | controlled clinical trial.pt. | 95074 |
| 8 | randomized.ab. | 396162 |
| 9 | placebo.ab. | 189436 |
| 10 | clinical trials as topic.sh. | 189502 |
| 11 | randomly.ab. | 280439 |
| 12 | trial.ti. | 175287 |
| 13 | or/6-12 | 1136737 |
| 14 | exp animals/ not humans.sh. | 4669475 |
| 15 | 13 not 14 | 1047097 |
| 16 | 5 and 15 | 1190 |
| 17 | limit 16 to (English or German) | 1142 |
| **Database:** EMBASE  **Platform:** Ovid  **Date of search:** November 292016  **Time Limits:** n/a  **Filters:** Line 6 - 15 are from the search filter: Cochrane search terms used to identify EMBASE  Reports of randomized trials for inclusion in CENTRAL; Ovid format. Available from:  http://handbook.cochrane.org/chapter_6/6_3_2_2_what_is_in_the_cochrane_central_  register_of_controlled.htm (accessed on October 2, 2015). | | |
| **#** | **Searches** | **Hits** |
| 1 | (formoterol or eformoterol or foradil or oxis or atimos modulite or atock or perforomist or salmeterol or serevent or tiotropium or spiriva or Ba 679 BR or indacaterol or onbrez or arcapta or NVA-237 or NVA237 or (NVA adj "237") or glycopyrronium or glycopyrrolate or seebri or enurev breezhaler or aclidinium or tudorza pressair or eklira genuair or symbicort or advair or seretide or olodaterol or striverdi or umeclidinium or GSK573719 or vilanterol or GW642444 or QVA149 or relovair or zephyr or anoro ellipta).ti,ab. | 10015 |
| 2 | exp Pulmonary Disease, Chronic Obstructive/ or exp Chronic obstructive lung disease/ | 98325 |
| 3 | (COPD or chronic obstructive pulmonary disease or COAD or chronic obstructive airway disease or chronic obstructive lung disease or chronic bronchitis or emphysema).ti,ab. | 110886 |
| 4 | 2 or 3 | 144702 |
| 5 | 1 and 4 | 4191 |
| 6 | (random$ or factorial$ or crossover$ or cross over$ or cross-over$ or placebo$ or (doubl$ adj blind$) or (singl$ adj blind$) or assign$ or allocat$ or volunteer$).ti,ab. | 1714581 |
| 7 | crossover-procedure/ or double-blind procedure/ or randomized controlled trial/ or single-blind procedure/ | 527528 |
| 8 | 6 or 7 | 1803906 |
| 9 | 5 and 8 | 2175 |
| 10 | limit 9 to (English or German) | 2120 |
| **Database:** Cochrane CENTRAL  **Platform:** Ovid  **Date of search:** 29 November 2016  **Time Limits:** n/a  **Filters:** n/a | | |
| **#** | **Searches** | **Hits** |
| 1 | (formoterol or eformoterol or foradil or oxis or atimos modulite or atock or perforomist or salmeterol or serevent or tiotropium or spiriva or Ba 679 BR or indacaterol or onbrez or arcapta or NVA-237 or NVA237 or (NVA adj "237") or glycopyrronium or glycopyrrolate or seebri or enurev breezhaler or aclidinium or tudorza pressair or eklira genuair or symbicort or advair or seretide or olodaterol or striverdi or umeclidinium or GSK573719 or vilanterol or GW642444 or QVA149 or relovair or zephyr or anoro ellipta).ti,ab,kw. | 5842 |
| 2 | exp Pulmonary Disease, Chronic Obstructive/ | 2618 |
| 3 | (COPD or chronic obstructive pulmonary disease or COAD or chronic obstructive airway disease or chronic obstructive lung disease or chronic bronchitis or emphysema).ti,ab,kw. | 12443 |
| 4 | 1 and (2 or 3) | 2349 |
| **Database:** Cochrane CDSR  **Platform:** Ovid  **Date of search:** 29 November 2016  **Time limits:** n/a  **Filters:** n/a | | |
| **#** | **Searches** | **Hits** |
| 1 | (formoterol or eformoterol or foradil or oxis or atimos modulite or atock or perforomist or salmeterol or serevent or tiotropium or spiriva or Ba 679 BR or indacaterol or onbrez or arcapta or NVA-237 or NVA237 or (NVA adj3 "237") or glycopyrronium bromide or glycopyrrolate or seebri or enurev breezhaler or aclidinium bromide or tudorza pressair or eklira genuair or symbicort or advair or seretide or olodaterol or striverdi or umeclidinium or GSK573719 or vilanterol or GW642444 or QVA149 or relovair or zephyr or anoro ellipta).ti,ab,kw. | 49 |
| 2 | (COPD or chronic obstructive pulmonary disease or COAD or chronic obstructive airway disease or chronic obstructive lung disease or chronic bronchitis or emphysema).ti,ab,kw. | 146 |
| 3 | 1 and 2 | 25 |
| **Database:** Dare  **Platform:** Ovid  **Date of search:** 29 November 2016  **Time Limits:** n/a  **Filters:** n/a | | |
| **#** | **Searches** | **Hits** |
| 1 | (formoterol or eformoterol or foradil or oxis or atimos modulite or atock or perforomist or salmeterol or serevent or tiotropium or spiriva or Ba 679 BR or indacaterol or onbrez or arcapta or NVA-237 or NVA237 or (NVA and "237") or glycopyrronium bromide or glycopyrrolate or seebri or enurev breezhaler or aclidinium bromide or tudorza pressair or eklira genuair or symbicort or advair or seretide or olodaterol or striverdi or umeclidinium or GSK573719 or vilanterol or GW642444 or QVA149 or relovair or zephyr or anoro ellipta).ti,kw,ft. | 82 |
| 2 | (COPD or chronic obstructive pulmonary disease or COAD or chronic obstructive airway disease or chronic obstructive lung disease or chronic bronchitis or emphysema).ti,kw,ft. | 397 |
| 3 | 1 and 2 | 47 |

Cochrane CENTRAL, Cochrane Central Register of Controlled Trials; Cochrane CDSR, Cochrane Database of Systematic Reviews; DARE, Database of Abstracts of Reviews of Effects; n/a, not applicable.

## Table S2: Eligibility criteria used in the systematic literature review

|  |  | Inclusion criteria | Exclusion criteria |
| --- | --- | --- | --- |
| **Study design** | **Abstract selection** | - Randomized controlled trials | - Cross-over studies, if cross over before 8 weeks in each arm - Post hoc or retrospective analyses (interested in moderate/severe subgroups) - Cost-effectiveness analyses - Observational studies - Reviews or meta-analyses - Methodology studies or protocols - N of 1 trials (sample size of 1 patient) - Studies lasting less than 8 weeks - Conference abstracts <2009 - Studies not in English |
| **Population** | **Full-text selection** | - Randomized controlled trials | - Studies where patients were required to spend time in a sleep laboratory |
|  | **Abstract selection** | - Patients with COPD as defined by GOLD guidelines (i.e. airflow limitation that is not fully reversible) - Studies that include asthma patients and COPD patients and report data for COPD patients separately - Adults - Studies that include adults and children and report data for adults separately - Subgroup data for moderate and severe COPD also of interest | - Studies with only healthy patients without COPD - Studies with patients who have reversible airway or obstructive lung disease - Studies with only patients with asthma - Studies that include asthma patients and COPD patients but do not report data for COPD patients separately - Studies with only patients who have alpha-1-antitrypsin-definciency-related COPD - Studies that include only children - Studies that include adults and children but do not report data for adults separately |
|  | **Full-text selection** | - Patients with COPD as defined by GOLD guidelines (i.e. airflow limitation that is not fully reversible) - Studies that include asthma patients and COPD patients and report data for COPD patients separately - Adults - Studies that include adults and children and report data for adults separately | - Studies with only healthy patients without COPD - Studies with patients who have reversible airway or obstructive lung disease - Studies with only patients with asthma - Studies that include asthma patients and COPD patients but do not report data for COPD patients separately - Studies with only patients who have alpha-1-antitrypsin-definciency-realted COPD - Studies that include only children - Studies that include adults and children but do not report data for adults separately |
| **Intervention** | **Abstract and full-text selection** | - LAMA/LABA combinations (US-approved dosages): - Umeclidinium/Vilanterol 62.5/25 μg OD (Anoro) - Olodaterol/tiotropium 5/5 μg OD (Stiolto) - Glycopyrrolate/formoterol 18/9.6 μg BID (Bevespi) - Glycopyrronium/indacaterol 15.6/27.5 μg BID (Utibron) | - Beta-agonists (bambuterol; fonoterol; tulobuterol) - Short-acting anticholinergics (Ipratropium; oxitropium) - Methylxanthines (theophylline) - leukotriene receptor antagonists (montelukast) - COPD drugs in development or targeting other pathways (roflumilast; polyvalent mechanical bacterial lysate; lipopolysaccharide) - All other pharmaceutical interventions not treating COPD (enoxaparin sodium) - Non-pharmaceutical interventions such as pulmonary rehabilitation |
| **Comparator** | **Abstract and full-text selection** | - Studies that compare treatments of interest (above) to: - Tiotropium 5 μg (Respimat) - Tiotropium 18 μg (Handihaler) | - Studies that only compare treatments that are not of interest - Studies that only include the treatments of interest in combination with treatments not of interest (i.e. prednisolone + formoterol) - Studies that only include the partial combinations of treatments of interest (i.e. tiotropium+ ICS) |
| **Outcomes** | **Abstract selection** | - No selection based on outcomes | - No selection based on outcomes |
|  | **Full-text selection** | - Report results for at least one of the following outcomes (for all treatments): - FEV_1_ trough; change from baseline - FEV_1_ peak; change from baseline - AUC FEV_1_; change from baseline - SGRQ score; change from baseline - SGRQ score; responder rate - Rescue medication; change from baseline - Adverse events; n (%) - Serious adverse events; n (%) - Outcomes should be reported at 8–16 weeks or 20–28 weeks. - Subgroup data for moderate and severe COPD also of interest | - None of the relevant outcomes (as listed in the inclusion criteria) is reported - Only report the following outcomes (without any outcomes of interest): - mortality; - bioactivity outcomes or biomarkers of inflammation; - lung mucociliary clearance; - arterial blood gases or degree of pulmonary hyper-inflation; - plethysmography and oscillometry; - nocturnal hypoxemia; - quality of life in EuroQol - Outcomes reported at other time points |

AUC, area under curve; BID, twice daily; COPD, chronic obstructive pulmonary disease; FEV_1_, forced expiratory volume in 1 second; GOLD, global initiative for chronic obstructive lung disease; ICS, inhaled corticosteroids; LABA, long-acting beta agonist; LAMA, long-acting muscarinic antagonist; OD, once daily; SGRQ, St. Georges Respiratory Questionnaire; US, United States of America.

## Table S3: Study design and patient characteristics identified from the systematic literature review (arms of interest only)

| **Study** | **Inclusion criteria** | **Additional COPD treatment** | **Treatments (ITT)** | **Trial**  **duration (weeks)** | **Male**  **(%)** | **Age, y**  **(SD)** | **Current smoker**  **(%)** | **Severe or very severe^a^**  **(%)** | **ICS use**  **(%)** | **Pack years (SD)** | **Mean FEV_1_^b^ (L)** | **FEV_1_ % predicted (SD)** |
| --- | --- | --- | --- | --- | --- | --- | --- | --- | --- | --- | --- | --- |
| **DB2113360^2^** | Outpatient; ≥40 years old; diagnosed with moderate-to- very severe COPD, as defined by the ATS-ERS; post-salbutamol FEV_1_/FVC ratio of <0.70 and a post-salbutamol FEV_1_ of ≤70%; current or former smoker, with a smoking history ≥10 pack-years; score of ≥2 on the mMRC Dyspnea Scale at study Visit 1. | Allowed: ICS at a dose of up to 1000 mcg/day of FP or equivalent, salbutamol/albuterol as rescue.  Not allowed: LABAs, SABAs, short acting anticholinergics and SABA/ICS combination products. | UMEC/VI; 62.5/25 μg; OD (n=212) | 24 | 69.8 | 63 (8.7) | 46.2 | 50.5 | 43.9 | 44.8 (27.7) | 1.31 (0.487) | 48 (12.9) |
|  |  |  | TIO; 18 μg; OD (n=208) |  | 67.3 | 62.6 (9.4) | 47.6 | 52.9 | 44.7 | 41.9 (24.4) | 1.30 (0.502) | 47.8 (13.4) |
| **DB2113374^2^** | Outpatient; ≥40 years old; diagnosed with moderate-to-very severe COPD, as defined by the ATS-ERS; post-salbutamol FEV_1_/FVC ratio of <0.70 and a post-salbutamol FEV_1_ of ≤70%; current or former smoker, with a smoking history ≥10 pack-years; score of ≥2 on the mMRC Dyspnea Scale at study Visit 1. | Allowed: ICS at a dose of up to 1000 mcg/day of FP or equivalent, salbutamol/albuterol as rescue.  Not allowed: LABAs, oral SABAs and LABAs, inhaled SABAs, inhaled short acting anticholinergics and SABA/ICS combination products. | UMEC/VI; 62.5/25 μg; OD (n=217) | 24 | 64.5 | 65.0 (8.6) | 42.4 | 50.7 | 47.5 | 47.8 (26.1) | 1.17 (0.466) | 47.7 (13.5) |
|  |  |  | TIO; 18 μg; OD (n=215) |  | 71.2 | 65.2 (8.3) | 47.4 | 51.6 | 53.5 | 54.0 (31.6) | 1.18 (0.429) | 47.4 (13.1) |
| **ZEP117115^3^** | Outpatient; ≥40 years old; diagnosed with moderate-to-very severe COPD and an established clinical history of COPD as defined by ATS/ERS, pre- and post-albuterol/salbutamol FEV_1_ ≤70% and a pre- and post-albuterol/salbutamol FEV_1_/FVC ratio <0.7. Current or former smokers with a smoking history of ≥10 pack-years. A score of ≥2 on the mMRC Dyspnea Scale at Visit 1. | Allowed: ICS at a dose of up to 1000 mcg/day of FP or equivalent, salbutamol/albuterol as rescue.  Not allowed: LABAs, SABAs, short acting anticholinergics and SABA/ICS combination products. | UMEC/VI; 62.5/25 μg; OD (n=454) | 24 | 68.3 | 61.9 (8.4) | 59.5 | 59.3 | 54.4 | 44.1 (24.4) | 1.26 (0.460) | 46.2 (13.0) |
|  |  |  | TIO; 18 μg; OD (n=451) |  | 67.2 | 62.7 (8.5) | 53.7 | 57.9 | 52.5 | 44.4 (25.0) | 1.26 (0.477) | 46.5 (12.8) |
| **DB2116960^4^** | ≥40 years old; diagnosed with moderate COPD, as defined by the ATS-ERS; post-salbutamol FEV_1_ of ≤70% and ≥50% of normal predicted values; mMRC Dyspnea Scale score of ≥1 at screening; in addition, patients were prescribed TIO for at least 3 months prior to screening. Patients were eligible for randomization after the 4-week run-in period if they had an mMRC score ≥1 at randomization, did not experience moderate-to-severe exacerbations or lower respiratory tract infections requiring antibiotic treatment between screening and randomization, and had treatment compliance with TIO of ≥80% and ≤120% during the run-in period. | Exclusion criteria at screening included the use of ICS or maintenance COPD medications other than TIO in the 3 months prior to screening (including other LAMAs, LABAs, LAMA/LABA combinations, ICS/LABA combinations, phosphodiesterase-4 inhibitors, theophyllines, and oral β_2_-agonists). Study supplied inhaled albuterol/salbutamol for use as relief medication throughout run-in and the treatment period, but must be withheld for at least 4 hours prior to spirometry testing | UMEC/VI; 62.5/25 μg; OD (n=247) | 12 | 66.0 | 64.5 (8.7) | 52.2 | 11.3 | NR | 38.6 (20.5) | NR | 59.8 (5.5) |
|  |  |  | TIO; 18 μg; OD (n=247) |  | 64.8 | 64.3 (8.7) | 47.8 | 13.8 | NR | 40.4 (20.2) | NR | 59.4 (5.3) |
| **PINNACLE 1^5^** | 40– 80 years old; diagnosed with moderate-to- very severe COPD, as defined by the ATS-ERS; post-bronchodilator FEV_1_/FVC ratio of <0.70 and a post-bronchodilator FEV_1_ of <80% predicted; Patients with FEV_1_ <30% predicted were required to have postbronchodilator FEV_1_ ≥750 mL; Current or former smoker, with a smoking history ≥10 pack-years; Dyspnea and disease burden were assessed (Visit 2) using the mMRC Dyspnea Scale and the COPD Assessment Test, respectively; these assessments were not used as entry criteria. | During the screening period, previously prescribed inhaled medications were stopped and patients were provided with open-label ipratropium bromide and open-label albuterol (as required up to four times daily) to control COPD symptoms. Ipratropium bromide was discontinued after randomization, whilst rescue albuterol was continued throughout the study as needed. Patients established (≥3 months) on a stable dose of oral corticosteroid, equivalent to ≤5 mg/day or ≤10 mg every other day, of prednisone could continue treatment. Patients using inhaled corticosteroids ICS) and/or phosphodiesterase-4 inhibitors at screening could continue using these as prescribed throughout the study. However, if an ICS was administered as part of an FDC, this was discontinued and substituted with an ICS single agent (fluticasone, mometasone, or budesonide) at the equivalent dose. Other prohibited medications included theophylline (>400 mg/day), leukotriene antagonists, mast-cell stabilizers, non-selective β-blockers, antiarrhythmic agents, antipsychotics and antidepressants (except selective serotonin [or serotonin–norepinephrine] reuptake inhibitors). | GLY/FOR; 18/9 μg; BID (n=526) | 24 | 55.1 | 62.6 (8.4) | 53.4 | 46.0 | 33.7 | 50.9 (26.8) | NR | 51.4 (13.6) |
|  |  |  | TIO; 18 μg; OD (n=451) |  | 59.6 | 63.0 (8.6) | 52.8 | 47.2 | 36.4 | 53.0 (27.5) | NR | 51.4 (13.8) |
| **TONADO 1^6^** | Outpatients aged ≥40 years with a history of moderate-to- very severe COPD (GOLD 2–4); post-bronchodilator FEV_1_ <80% of predicted normal; post-bronchodilator FEV_1_/ FVC <70%; current or ex-smokers with a smoking history of >10 pack-years. | Patients continued to receive treatment with inhaled corticosteroids as required and were provided with salbutamol/albuterol metered-dose inhaler (100 μg per actuation) as rescue medication to be used as necessary at any point during the trial. Temporary increases in the dose or addition of oral steroids or theophylline preparations were allowed during the treatment portion of the study; PFTs were not performed within 7 days of the last administered dose. | TIO/OLO; 5/5 μg; OD (n=522) | 52 | 73.6 | 64.8 (8.2) | 36.2 | 50.6 | 51.7 | NR | 1.17 (0.47) | 49.5 (15.2) |
|  |  |  | TIO; 5 μg; OD (n=527) |  | 72.7 | 64.2 (8.5) | 35.7 | 50.1 | 45.0 | NR | 1.20 (0.50) | 49.7 (15.3) |
| **TONADO 2^6^** | Outpatients aged ≥40 years with a history of moderate-to- very severe COPD (GOLD 2–4); post-bronchodilator FEV_1_ <80% of predicted normal; post-bronchodilator FEV_1_/ FVC <70%; current or ex-smokers with a smoking history of >10 pack-years. | Patients continued to receive treatment with ICS as required and were provided with salbutamol/albuterol metered-dose inhaler (100 μg per actuation) as rescue medication to be used as necessary at any point during the trial. Temporary increases in the dose or addition of oral steroids or theophylline preparations were allowed during the treatment portion of the study; PFTs were not performed within 7 days of the last administered dose. | TIO/OLO; 5/5 μg; OD (n=507) | 52 | 68.8 | 62.7 (8.4) | 41.6 | 51.8 | 46.5 | NR | 1.19 (0.51) | 49.1 (15.4) |
|  |  |  | TIO; 5 μg; OD (n=506) |  | 73.5 | 63.5 (8.7) | 36.0 | 49.6 | 45.3 | NR | 1.20 (0.51) | 49.7 (16.1) |
| **OTEMTO 1^7^** | ≥40 years of age with moderate-to-severe COPD (GOLD 2-3); post-bronchodilator FEV_1_ ≥30 and <80% of predicted normal; FEV_1_/FVC <70% predicted and a smoking history >10 pack-years | Patients were allowed to continue their ICS therapy (if they were on a stable dose for 6 weeks prior to screening). LAMAs or LABAs other than study medication were prohibited during the screening or treatment periods, and short-acting muscarinic antagonists were permitted only during the screening period. Open-label salbutamol was provided as rescue medication for use throughout the study. | TIO/OLO; 5/5 μg; OD (n=203) | 12 | 56.2 | 64.7 (8.9) | 54.7 | 36.0 | 41.9 | NR | 1.32 (0.491) | 54.9 (12.0) |
|  |  |  | TIO; 5 μg; OD (n=203) |  | 61.1 | 64.9 (8.2) | 48.3 | 37.0 | 37.9 | NR | 1.31 (0.458) | 54.7 (12.8) |
| **OTEMTO 2^7^** | ≥40 years of age with moderate-to-severe COPD (GOLD 2-3); post-bronchodilator FEV_1_ ≥30 and <80% of predicted normal; FEV_1_/FVC <70% predicted and a smoking history >10 pack-years | Patients were allowed to continue their ICS therapy (if they were on a stable dose for 6 weeks prior to screening). LAMAs or LABAs other than study medication were prohibited during the screening or treatment periods, and short-acting muscarinic antagonists were permitted only during the screening period. Open-label salbutamol was provided as rescue medication for use throughout the study. | TIO/OLO; 5/5 μg; OD (n=202) | 12 | 65.8 | 65.2 (8.5) | 45.5 | 38.1 | 35.6 | NR | 1.36 (0.467) | 54.8 (12.8) |
|  |  |  | TIO; 5 μg; OD (n=203) |  | 64.0 | 64.7 (8.4) | 44.8 | 32.5 | 35.0 | NR | 1.40 (0.511) | 55.9 (12.2) |

^a^Calculated severe and very severe patients; ^b^pre-bronchodilator

ATS, American Thoracic Society; BID, twice daily; COPD, chronic obstructive pulmonary disease; ERS, European Respiratory Society; FDC, fixed dose combination; FEV_1_, forced expiratory volume in 1 second; FOR, formoterol; FP, fluticasone propionate; FVC, forced vital capacity; GLY, glycopyrrolate; GOLD, global initiative for chronic obstructive lung disease; ICS, inhaled corticosteroids; ITT, intent-to treat population; LABA, long acting β_2_-agonists; LAMA; long-acting muscarinic antagonists; mMRC, modified Medical Research Council; NR, not reported; OD, once daily; OLO, olodaterol; PFT, pulmonary function test; SABA, short acting β_2_-agonists; SD, standard deviation; TIO, tiotropium; UMEC, umeclidinium; VI, vilanterol.

## Table S4: Heterogeneity analysis and model choice used in the meta-analysis

| Endpoints | Time point, week | I^2^, % | p value | Meta-analysis model |
| --- | --- | --- | --- | --- |
| FEV_1_ trough | 12 | 71 | <0.01 | Random effects model |
|  | 24 | 74 | <0.01 | Random effects model |
| FEV_1_ peak | 12 | 0 | 0.67 | Fixed effect model |
|  | 24 | 0 | 0.88 | Fixed effect model |
| FEV_1_ AUC | 12 | 34 | 0.21 | Fixed effect model |
| SGRQ responder rate | 12 | 0 | 0.60 | Fixed effect model |
|  | 24 | 49 | 0.12 | Fixed effect model |
| SGRQ total score | 12 | 0 | 0.71 | Fixed effect model |
|  | 24 | 23 | 0.27 | Fixed effect model |
| Rescue medication use | 24 | 0 | 0.62 | Fixed effect model |
| AE | 24 | 38 | 0.18 | Fixed effect model |
|  | 24 and 52 (exploratory) | 14 | 0.32 | Fixed effect model |
| SAE | 24 | 58 | 0.07 | Fixed effect model |
|  | 24 and 52 (exploratory) | 43 | 0.12 | Fixed effect model |

AE, adverse event; AUC, area under curve; FEV1, forced expiratory volume in 1 second; SAE, serious adverse event; SGRQ, St. Georges Respiratory Questionnaire.

## Table S5: Individual study results at 12 and 24 weeks

|  |  | DB2113360^2^ | DB2113374^2^ | ZEP117115^3^ | PINNACLE 1^5^ | TONADO 1^6^ | TONADO 2^6^ | OTEMTO 1^7^ | OTEMTO 2^8^ |
| --- | --- | --- | --- | --- | --- | --- | --- | --- | --- |
|  |  | UMEC/VI  62.5/25 μg vs  TIO 18 μg | UMEC/VI 62.5/25 μg vs TIO 18 μg | UMEC/VI 62.5/25 μg vs TIO 18 μg | GLY/FOR 18/9.6 μg BID vs TIO 18 μg | TIO/OLO 5/5 μg vs TIO 5 μg | TIO/OLO 5/5 μg vs TIO 5 μg | TIO/OLO 5/5 μg vs TIO 5 μg | TIO/OLO 5/5 μg vs TIO 5 μg |
| Week 12 | ΔCFB in FEV_1_ trough (L), mean (SE) | 80.0 (24.5) | 95.0 (21.9) | 109.0 (15.8) | 24.0* (14.5)*‡ | 76.0* (18.8)¶ | 59.3* (18.8) ¶ | 28.0 (19.0) | 39.0 (19.0) |
|  | ΔCFB in FEV_1_ peak (L), mean (SE) | 63.0 (26.0) | 104.0 (23.5) | 95.0 (17.1) | 95.0* (22.2)§ | NR | NR | NR | NR |
|  | ΔCFB in FEV_1_ AUC_0–3_ (L), mean (SE) | NR | NR | NR | NR | 156.0* (19.0) | 135.0* (19.0) ¶ | 111.0 (19.0) | 105.0 (19.0) |
|  | SGRQ responder rate, n/N (%) | UMEC/VI:  103/183 (56.3)†  TIO:  92/173 (53.2)† | UMEC/VI: 111/189 (58.7)†  TIO:  99/187 (52.9)† | UMEC/VI: 244/454 (56.0)†  TIO:  199/451 (47.0)† | NR | NR | NR | TIO/OLO: 104/196 (53.1)  TIO:  80/192 (41.7) | TIO/OLO: 102/197 (51.8)  TIO:  79/192 (41.1) |
|  | ΔCFB in SGRQ total score, mean (SE) | -0.2 (1.3) | -2.0 (1.3) | -2.1 (0.7) | NR | NR | NR | -2.5 (1.0) | -1.7 (1.0) |
| Week 24 | ΔCFB in FEV_1_ trough, (L), mean (SE) | 90.0 (26.0) | 60.0 (25.3) | 112.0 (16.1) | 21.0 (15.3) | 71.0 (12.0) | 50.0 (13.0) | NR | NR |
|  | ΔCFB in FEV_1_ peak (L), mean (SE) | 72.0 (27.0) | 93.0 (25.0) | 95.0 (18.6) | 97.0 (15.3) | NR | NR | NR | NR |
|  | ΔCFB in FEV_1_ AUC_0–3_ (L), mean (SE) | NR | NR | NR | NR | 117 (12) | 103 (12) | NR | NR |
|  | SGRQ responder rate, n/N (%) | UMEC/VI:  94/193 (48.7)†  TIO:  92/178 (51.7)† | UMEC/VI: 103/190 (54.2)†  TIO:  104/190 (54.7)† | UMEC/VI: 237/454 (53.0)  TIO:  196/451 (46.0) | GLY/FOR:  198/526 (37.6)†  TIO:  173/451 (38.4)† | NR | NR | NR | NR |
|  | ΔCFB in SGRQ total score, mean (SE) | 0.8 (1.5) | -0.2 (1.4) | -2.1 (0.8) | -0.7 (0.8) | NR | NR | NR | NR |
|  | ΔCFB in RMU, puffs/day, mean (SE) | -0.7 (0.3) | -0.6 (0.3) | -0.5 (0.1) | -0.3 (0.1) | NR | NR | NR | NR |
|  | AEs, n (%) | UMEC/VI: 108 (50.9)†  TIO: 82 (39.4)† | UMEC/VI: 127 (58.5)†  TIO: 126 (58.6)† | UMEC/VI: 202 (44.5)†  TIO: 190 (42.1)† | GLY/FOR: 331 (62.9)  TIO: 283 (62.7) | TIO/OLO: 387 (74.1)**  TIO: 381 (72.3)** | TIO/OLO: 374 (73.8)**  TIO: 376 (74.3)** | NR | NR |
|  | SAEs, n (%) | UMEC/VI: 7 (3.3)†  TIO: 13 (6.3)† | UMEC/VI: 22 (10.1)†  TIO: 9 (4.2)† | UMEC/VI: 16 (3.5)†  TIO: 17 (3.8)† | GLY/FOR: 44 (8.4)  TIO: 36 (8.0) | TIO/OLO: 87 (16.7)**  TIO: 79 (15.0)** | TIO/OLO: 82 (16.2)**  TIO: 93 (18.4)** | NR | NR |

*ΔCFB calculated using CFB outcomes; †percentage calculated; ‡legend of figure in manuscript was incorrect, with corrected values presented here; ¶based on imputed SE values (average) for CFB; §based on imputed SE values (average) for CFB; **only included in exploratory analysis (AEs reported at 52 weeks)

AE, adverse event; AUC, area under curve; ΔCFB, difference of change from baseline; FEV_1_, forced expiratory volume in 1 second; FOR, formoterol; GLY, glycopyrrolate; NR, not reported; OLO, olodaterol; RMU, rescue medication use; SAE, serious adverse event; SGRQ, St. Georges Respiratory Questionnaire; TIO, tiotropium; UMEC, umeclidinium.

## Figure S1: Flow chart of study selection process


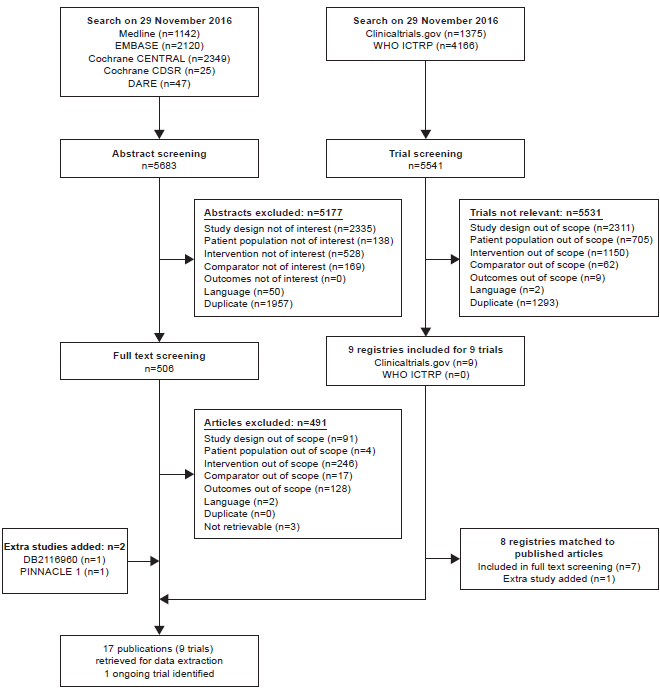


## CDSR, Cochrane Database of Systematic Reviews; CENTRAL, Central Register of Controlled Trials, DARE, Database of Abstracts of Reviews of Effects; ICTRP, International Clinical Trials Registry Platform; WHO, World Health Organization.

## Figure S2: Forest plots of LAMA/LABA versus TIO for AEs and SAEs at 24 weeks (expoloratory analysis)


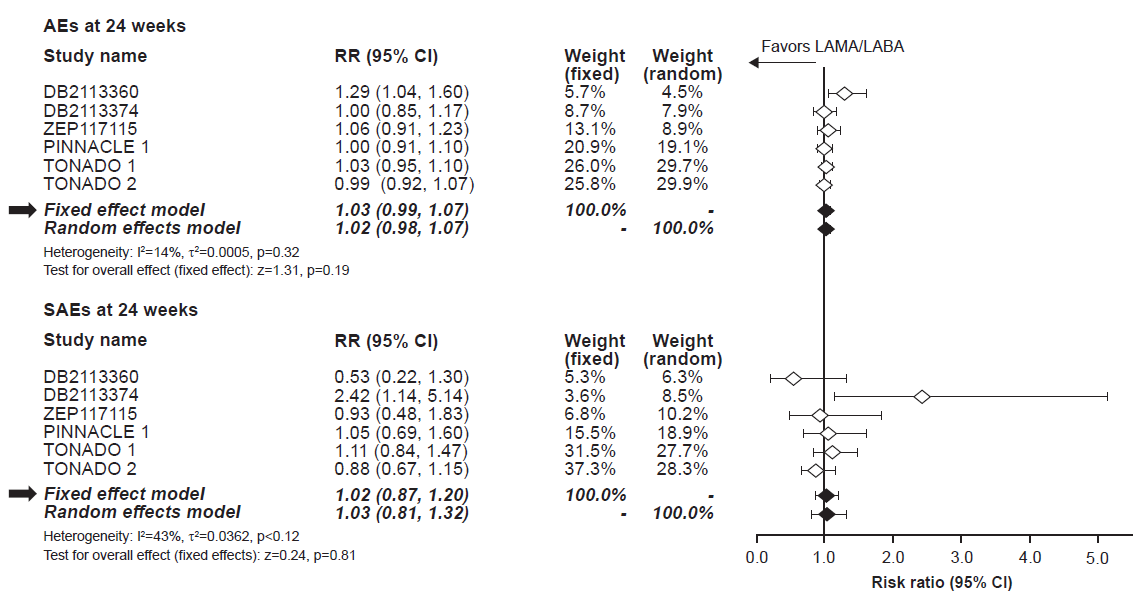


AE, adverse event; CI, confidence interval; I^2^, proportion of variability across trials due to heterogeneity; LABA, long-acting beta agonist; LAMA, long-acting muscarinic antagonist; RR, risk ratio; SAE, serious adverse event; SE, standard error; τ^2^, between study variance in random effects meta-analysis; TIO, tiotropium.

# References

**1.** Higgins JPT, Green S & (editors). Cochrane Handbook for Systematic Reviews of Interventions Version 5.1.0 [updated March 2011], [www.handbook.cochrane.org](http://www.handbook.cochrane.org)

**2.** Decramer, M., Anzueto, A., Kerwin, E., Kaelin, T., Richard, N., et al. Efficacy and safety of umeclidinium plus vilanterol versus tiotropium, vilanterol, or umeclidinium monotherapies over 24 weeks in patients with chronic obstructive pulmonary disease: results from two multicentre, blinded, randomised controlled trials. *The Lancet Respiratory Medicine.* **2**, 472-486 (2014).

**3.** Maleki-Yazdi MR, Kaelin T, Richard N, Zvarich M, Church A. Efficacy and safety of umeclidinium/vilanterol 62.5/25 mcg and tiotropium 18 mcg in chronic obstructive pulmonary disease: results of a 24-week, randomized, controlled trial. *Respir Med.* **108**, 1752-1760 (2014).

**4.** Kerwin, E.M., Kalberg, C.J., Galkin, D.V., Zhu, C.Q., Church, A., et al. Umeclidinium/vilanterol as step-up therapy from tiotropium in patients with moderate COPD: a randomized, parallel-group, 12-week study. *Int J COPD.* **12**, 745-755 (2017).

**5.** Martinez, F.J., Rabe, K.F., Ferguson, G.T., Fabbri, L.M., Rennard, S., et al. Efficacy and Safety of Glycopyrrolate/Formoterol Metered Dose Inhaler Formulated Using Co-Suspension Delivery Technology in Patients With COPD. *Chest.* **150**, 340-357 (2017).

**6.** Buhl, R., Maltais, F., Abrahams, R., Biermer, L., Derom, E., et al. Tiotropium and olodaterol fixed-dose combination versus mono-components in COPD (GOLD 2-4). *Eur Respir J.* **45**, 969-979 (2015).

**7.** Singh, D., Ferguson, G.T., Bolitschek, J., Gronke, L., Hallmann, C., et al. Tiotropium + olodaterol shows clinically meaningful improvements in quality of life. *Respir Med.* **109**, 1312-1319 (2015)
